# Responses in adult pied flycatcher males depend on playback song similarity to local population

**DOI:** 10.1093/beheco/arae090

**Published:** 2024-11-06

**Authors:** Mario Gallego-Abenza, Fanny-Linn H Kraft, Lan Ma, Samyuktha Rajan, David Wheatcroft

**Affiliations:** Department of Zoology, Stockholm University, Svante Arrhenius väg, 106 91 Stockholm, Sweden; Department of Zoology, Stockholm University, Svante Arrhenius väg, 106 91 Stockholm, Sweden; Department of Zoology, Stockholm University, Svante Arrhenius väg, 106 91 Stockholm, Sweden; Department of Zoology, Stockholm University, Svante Arrhenius väg, 106 91 Stockholm, Sweden; Department of Zoology, Stockholm University, Svante Arrhenius väg, 106 91 Stockholm, Sweden

**Keywords:** Acoustic dissimilarity, Sexual signals divergence, Song complexity, Song discrimination

## Abstract

Song divergence driven by social learning has been proposed to be a key factor driving allopatric speciation in oscine birds. Songbirds often respond more to songs deriving from their local population, suggesting the potential for acoustic divergence across populations to shape both intra- and intersexual interactions. However, many of these studies were conducted on species with simple songs and, as a result, we know comparatively little about the emergence of population differences and song discrimination in species with complex songs. We addressed this question in the pied flycatcher (*Ficedula hypoleuca*) by calculating the dissimilarity of songs from 2 foreign populations as well as from our study site to the local centroid. We then conducted a paired-design playback experiment where both local and foreign songs were played simultaneously. We found that pied flycatcher males showed significantly stronger responses to those songs that sounded more similar to the local population. This suggests that despite the high complexity of the pied flycatcher song, individuals are still able to discriminate across populations. Our results support the hypothesis that learned song divergence can act as a mechanism for assortative mating and allopatric speciation.

## Teaser

Differences in sexual advertisements across populations, like bird song, can hinder effective communication. However, the buildup of differences and, therefore, barriers to communication are thought to be delayed when songs are highly complex. Here, we find that complex-singing pied flycatcher males respond more strongly to songs that are more similar to those from their own population. This suggests the emergence of song differences is substantial enough to impact communication and potentially affect their mating interactions despite complex song.

## Introduction

Understanding how sexual selection shapes phenotypic divergence among populations has been a long-term focus among evolutionary biologists (Schluter 2000; Coyne and Orr 2004; Price 2008). In particular, divergence in sexual signals is thought to drive reproductive isolation among populations and, subsequently, speciation (Gray and Cade 2000). The buildup of divergence across populations is thought to be faster when sexual signals are acquired through social learning. A classic example is songbird song, a learned sexually selected trait for which differences across populations within the same species are ubiquitous (Catchpole and Slater 2008a). Social learning leads to cultural evolution within populations (Derryberry 2007; Mennill et al. 2018), which is typically thought to speed up the rate of divergence across populations relative to genetically inherited signals, due to combination of higher mutation rates and social conformity (Lachlan et al. 2004; Edwards et al. 2005; Mason et al. 2017). However, a direct role of social learning in the emergence of population differences and speciation remains controversial (Slabbekoorn and Smith 2002; Lachlan and Servedio 2004; Freeman et al. 2017; Mason et al. 2017)

Song discrimination at the population level has been demonstrated in a variety of songbird species (reviewed in Parker et al. 2018). However, the generation of population-level song differences and, therefore, also of song discrimination is thought to be challenged when songs contain a wider range of unique sounds (Goodfellow and Slater 1986; Williams and Slater 1990; Catchpole and Slater 2008a), which might drive broad, nonspecific responses in receivers (Slabbekoorn and Smith 2002; Freeman et al. 2017). The range of unique sounds (i.e. “syllables”) produced is one of the typical measures of song complexity and one that, at least in some species, is associated with female preferences (Catchpole and Slater 2008a). In many of the species in which individuals express population-level song discrimination, males sing relatively stereotyped simple songs, where discrete changes in song sub-units (i.e. syllables) can delimitate song boundaries across populations (i.e. song “dialects”). For example, song discrimination has been classically studied in white-crowned sparrows (*Zonotrichia leucophrys*), whose songs contain between 3 and 6 syllable types (Milligan and Verner 1971; Orejuela and Morton 1975; Baker 1982; Thompson and Baker 1993; Macdougall-Shackleton and Macdougall-Shackleton 2001). Other examples include Darwin´s ground finches with songs of 1-4 unique syllables (Ratcliffe and Grant 1985; Podos and Warren 2007), Darwin’s mangrove finch (*Camarhynchus heliobates*), with 1 syllable (Brumm et al. 2010), and rufous-collared sparrows (*Zonotrichia capensis*), with 2 to 4 unique syllables (Danner et al. 2011). Neighbour-stranger discrimination, like population-level discrimination, has been hypothesized to be more difficult in species with larger repertoires (e.g. Kroodsma 1976). However, comparative studies have suggested little relationship between the strength of neighbor–stranger discrimination and repertoire size (Weary et al. 1992; Moser-Purdy and Mennill 2016). To the best of our knowledge, only one study demonstrated both formation and discrimination of song dialects despite highly complex songs, in skylarks (*Alauda arvensis*) (Briefer et al. 2008), where a single male can sing up to 700 different syllables (Aubin 1981). As a result, additional work is needed to help elucidate whether (or to what degree) song complexity hinders the buildup of song discrimination at the population level.

Songbird song is socially learned within genetically determined constraints, which influence the types of songs likely to be learned (Catchpole and Slater 2008b). This combination of genetic constraints and learning determine both song production in males and song preferences in females. For instance, juvenile male song sparrows (*Melospiza melodia*) that are exposed to both conspecific and heterospecific songs during early life selectively learn conspecific songs (Marler and Peters 1977). Such genetic predispositions may even guide learning onto specific dialects within species (Nelson 2000). However, within these species-specifics bounds, experience plays a critical role in what songs males sing (Clayton 1989; Eriksen et al. 2009; Mennill et al. 2018). Likewise, female songbirds acquire their song preferences by listening to the songs produced by adult males during development (Riebel 2000; Fujii et al. 2021) but are more likely to be stimulated by (Hauber et al. 2013) and/or learn (Baker et al. 1981) certain songs over others.

Ultimately, female responses are thought to determine the consequence of population-level song differences on reproductive isolation and, therefore, speciation (Price 2008). However, female song preferences remain challenging to assess, particularly in the wild, and, as a result, researchers often use song responses in male birds as a proxy for female responses (e.g. Freeman and Montgomery 2017; Freeman et al. 2017; Lipshutz et al. 2017). This approach is supported by the widespread observation that male songbirds respond to a wider range of song stimuli than do females (Baker 1982; Searcy et al. 1997; Danner et al. 2011; Anderson et al. 2014). Stronger song discrimination in females is thought to be due to larger fitness costs to females responding to inappropriate sexual traits, they risk pairing with poorly adapted males (Searcy and Brenowitz 1988) or hybridizing (Qvarnström et al. 2006). As a result, male discrimination between 2 types of song stimuli is assumed to imply female discrimination of the same stimuli.

Male songbirds respond to hearing songs in a variety of ways, but aggressive responses are particularly common during periods of the breeding season associated with resource defense or mate guarding (Catchpole and Slater 2008a), when failure to respond could mean loss of territory or paternity. Thus, males are expected to be tuned to songs that are associated with potential competitors and restrict their aggressive responses to those particularly likely to represent threats. In territorial species, male songbirds often display the strongest aggressive responses to songs from unfamiliar rather than familiar individuals, consistent with the “Dear Enemy” effect (Temeles 1994; Werba et al. 2022). Population-level discrimination in male birds is influenced by hearing local songs and, moreover, might arise as a byproduct of neighbor–stranger discrimination. However, discrimination of local songs has been shown to arise independently from familiarity with particular neighbors and, instead, depends on developmental auditory experience (Williams et al. 2024) and genetic factors (Nelson 2000; Wheatcroft et al. 2022).

In this study, we explore whether song differences between geographically distant breeding populations lead to song discrimination in the pied flycatcher (*Ficedula hypoleuca*) by comparing their responses to the songs of unfamiliar local and foreign males. Pied flycatchers are a migratory species that breeds across Europe and spends the winter in sub-Saharan Africa (Lundberg and Alatalo 1992). After arriving from wintering grounds, males compete over access to nesting holes and sing regularly near potential nesting sites to attract females (Lundberg and Alatalo 1992). Later in the breeding season, some males may sing on additional territories to attract secondary females and/or pursue extra-pair copulations (Lundberg and Alatalo 1992). Male pied flycatcher song typically consists of around 8 to 11 syllables, some of which are repeated (Lundberg and Alatalo 1992). Syllables can be arranged in different ways to generate a broad range of songs that vary across individuals (Lundberg and Alatalo 1992) and breeding populations (Haavie et al. 2004; Wheatcroft et al. 2022). Male syllable repertoires (i.e. the total number of unique syllables produced) are often larger than 50 syllables (Lampe and Saetre 1995; Eriksen et al. 2009). As a result, the pied flycatcher is a species with relatively complex song. According to a recent comparative study exploring the link between song complexity and cognition in 23 species with vocalization repertoire ranging from 4 to 337.5 (Audet et al. 2023), the pied flycatcher ranks fifth in vocal repertoire. Moreover, pied flycatchers are thought to have a relatively plastic song, able to acquire song syllables from conspecifics throughout their lives (Eriksen et al. 2011) and even copying the song of surrounding heterospecifics (Lundberg and Alatalo 1992; Haavie et al. 2004). Female pied flycatchers base their mate choice at least in part on variation in male song, preferring males that produce more complex songs (Lampe and Saetre 1995). Detectable song differences are apparent between populations located as little as 50 km apart (Wheatcroft et al. 2022). Despite the acoustic overlap in songs between populations, these differences are sufficient for discrimination by nestling birds, who respond most strongly to songs typical of their own population (Wheatcroft et al. 2022). We here aim to determine whether population-level song discrimination is also expressed into adulthood, even for species with complex song. To answer this, we conducted a paired-design playback experiment exposing adult males to both local and foreign songs. We predicted that adult males would show greater aggressive responses toward songs more similar to those from their local population.

## Material and methods

### Study site and species

This study took place in a monitored pied flycatcher population breeding in nest boxes in mainland Sweden (Tovetorp; 58°56ʹ46.5″N, 17°09ʹ19″E). Pied flycatchers breed in Europe and, as a hole nesting species, are well adapted to use nest boxes (Lundberg and Alatalo 1992). The breeding season begins with males arriving in late April and producing songs as a sexual display close to nest boxes or natural cavities to attract females (Eriksson and Wallin 1986; Lampe and Saetre 1995). Apart from attracting females, song production aids arriving males to acquire territories in suitable areas with conspecifics, as well as maintaining territory boundaries (Alatalo et al. 1982; Lundberg and Alatalo 1992). In both years (2022 to 2023), we conducted playback experiments over 2 continuous weeks, starting soon after males’ arrival from wintering grounds.

### Recordings and acoustic analyses of played-back songs

We utilized previously recorded songs of 10 individual males breeding at Tovetorp in 2020 as “local” playback stimuli, as used in Wheatcroft et al. (2022). Given the low returning rate of adult males over breeding years (e.g. out of 36 breeding males in 2020, only 1 individual bred in our study population in 2022), it is likely that breeding males in 2022 to 2023 were unfamiliar with the specific individuals whose songs we played back. To test males responses to a broad range of foreign song variation, we constructed playback stimuli using previous recorded songs from males from 2 distinct breeding populations, which together comprised our “foreign” treatment: La Hiruela, Spain, 41°4ʹN, 3°27´W (2,451 km from Tovetorp) (*N* = 13 individuals) and the Netherlands, 52°04ʹN, 5°49E (1,045 km from Tovetorp) (*N* = 11 individuals). Each 1-min playback contained a median of 10 unique strophes (range = 8 to 12) of a single individual separated by 4-s silence intervals at a standardized volume of 80 dB at 1 m distance (A-weighting, Sound Level meter: Velleman DEM202).

Acoustic measurements of the playbacks (571 songs) were compared using the built-in tool dynamic time-warping (DTW) included in Luscinia software (Lachlan 2022). In the DTW analyses, we included an additional 1,087 songs from 96 males (mean of songs per male = 11.3, range = 2 to 34) recorded in 4 other foreign populations: Lund, Sweden (55°40ʹN, 13°33ʹE), 40 males; Valsaín, Spain (40°52ʹN, 4°01ʹW), 15 males; Dartmoor, UK (50°36ʹN, 3°43ʹW) (12 males); and Drenthe, The Netherlands (52°49ʹN, 6°22ʹE), 29 males. Similarly, we included 72 songs from 5 males recorded in La Hiruela as well as 449 songs from 41 local males. These additional songs were included to provide a better representation of the acoustic features that vary across populations. The DTW analysis in Luscinia produces dissimilarity matrices for all songs for a range of acoustic features. These matrices are subjected to non-metric multidimensional scaling into 10 dimensions, followed by a principal components analysis. These song measures were subsequently averaged within playback file, explaining the 87.37% of the total variation in dissimilarity across songs. The resultant scores from all ten principal components were used to calculate the Euclidean distance of each played back file (including those containing songs of local Tovetorp males) to the mean centroid of the local Tovetorp population, obtaining thus the so-called “Dissimilarity to local centroid” used in our statistical analyses.

### Playback experiment protocol

We conducted a playback choice experiment by simulating simultaneous intruders in pairs of empty nest boxes, separated a median of 34.2 m (range = 11 to 68 m). This method has previously been used to measure song preference in our study species (Wheatcroft and Qvarnström 2017). The averaged minimum distance between trials conducted in the same day was 621.97 m (range = 124.2 to 1,673.5 m). This distance makes it extremely unlikely that the same individual was tested multiple times, helping to ensure the independence of trials. While setting up the equipment (5 to 10 min), experimenters reported whether a male was already present in the surroundings of any of the 2 nest boxes. This information was subsequently used as an explanatory factor (“Male seen”) to control for potentially biased responses in our statistical analyses due to the presence of a male in one or two of the treatments. Experimenters mounted a video camera (Mod. Raspberry Pi Camera module V2) at a distance of 1 m from the nest box’s entrance together with a wooden pied flycatcher dummy male attached to the box’s lid. In addition, they placed a loudspeaker (W-King D8 mini-1, frequency response: 100 Hz to 16 KHz) on the ground, ~1.5 m underneath the nest box. Treatments (local or foreign) were randomly assigned to each of the paired nest boxes, and within each treatment, playbacks were chosen randomly. Playbacks were started simultaneously from both speakers and played back for 1 h in the absence of experimenters.

### Behavioral responses

We used the free software Solomon Coder (https://solomon.andraspeter.com/) to score the behavioral responses of pied flycatcher males during each 1-h playback. We utilized 2 distinct measurements of each male’s response. First, the total amount of time that the male was present within the focal area of the camera, including the time inside the nest box. The resulting calculation could range from 0 to 3600 seconds, called hereafter “Time present,” and was rounded to the nearest whole second. Second, we assessed the male’s aggressive response, so-called “Aggressiveness,” by counting the number of times he pecked on the wooden dummy. Both response variables, “Time present” and “Aggressiveness,” showed a moderate positive correlation (Pearson correlation test: *r* = 0.538, *P* = < 0.0001). However, we analyzed them separately given that c.a. only a third of the responding males showed aggression toward the dummy. Videos were coded by a single observer (MG-A) who was blind to the acoustic similarity of played-back songs to the local dialect but was aware of the played-back songs’ origin. To account for potential observer bias, we conducted an inter-observer reliability test with a blind observer on a proportion of videos (4 videos, 7.4% of the total) and assessed their interrater agreement using the interclass correlation coefficient for continuous measurements, function “icc” (ICC = 1, *P* < 0.0001); package “irr” v. 0.84.1. (Gamer et al. 2019).

### Statistical analyses

We used R software (v R. 4.2) (R Core Team 2017) for all statistical analyses. Only experiments in which at least one of the 2 boxes were visited during the playbacks were included in the analyses. Both response variables “Time present” and “Aggressiveness” were treated as counts and modeled using the function “glmmTMB,” family “nbinom1” within the R package “glmmTMB” v. 1.1.4. (Brooks et al. 2017). In both cases, the family distribution was chosen based on AIC comparison, where the “nbinom1” distribution resulted in a lower AIC compared to the “Poisson” distribution. The differences in AIC were 1529 and 24.8 in “Time present” and “Aggressiveness,” respectively. AIC differences to assess model goodness have been suggested as a reliable tool in behavioral ecology (Bolker et al. 2012; Hilbe 2014). As predictors, we included “Dissimilarity to local centroid” (continuous), “Male seen” (Yes—No) and “Year” (2022 to 2023). We evaluated the significance of the predictors using likelihood-ratio tests comparing a model with each predictor to a model lacking each predictor, using the function “lrtest,” package “lmtest” v. 0.9.40. (Zeileis and Hothorn 2002). We included “Played back male” (males’ ID from which we composed our played-back stimuli) and “Trial ID” (paired playback choice experiment) as random factors in both models. The utilization of “Dissimilarity to local centroid” as a continuous predictor in our models was supported by AIC comparison to those models containing “Treatment” (Local—Foreign) (“Time present,” ΔAIC = 1.2; “Aggressiveness,” ΔAIC = 2.4). We ensured neither overdispersion nor zero-inflation in both models using the simulation test of “DHARMa” package v. 0.4.6. (Hartig 2022).

## Results

**Fig. 1.**
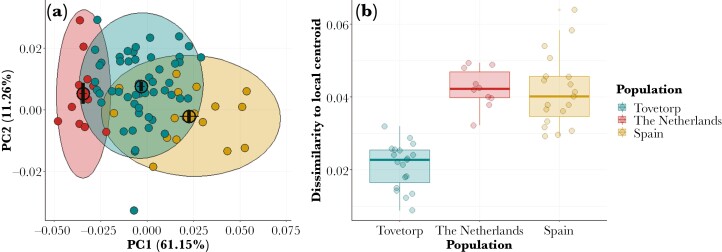
Acoustic analyses from foreign and local songs. (a) Variation in principal components of songs, PC1 and PC2 scores are averaged per song and individual. Apart from individuals used as playback stimuli, we added extra individuals from Tovetorp (41) and Spain (4) to represent a broader view of population-specific song variation. (b) Dissimilarity values to the local centroid of the played-back songs calculated as the Euclidean distance from the 10 principal components.

**Fig. 2.**
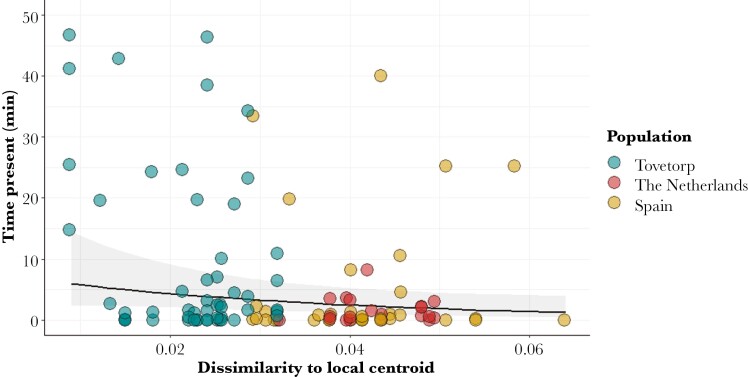
Scatterplot showing the influence of dissimilarity to local centroid of played-back songs on the duration of pied flycatcher males’ responses back-transformed to minutes. Solid line represents the overall linear fit with shaded areas representing the confidence intervals, extracted using the function “plot_model” in “sjPlot” v. 2.8.11 r package (Lüdecke 2024). Dots represent the observed data.

**Fig. 3.**
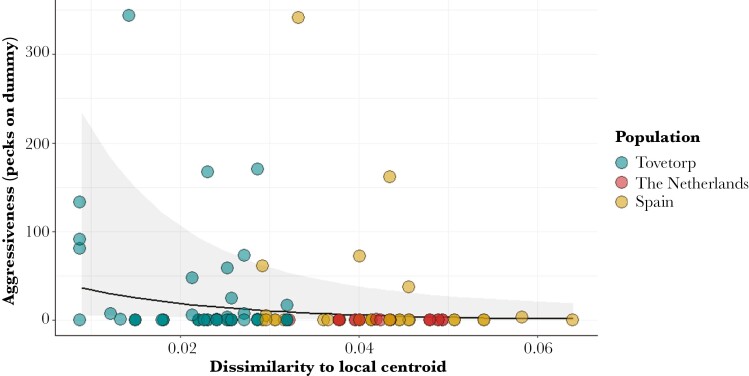
Scatterplot showing the influence of dissimilarity to local centroid of played-back songs on pied flycatcher males’ aggressive responses toward the wooden dummy. Solid line represents the predicted linear fit with shaded areas representing the confidence intervals, both were extracted using the function “plot_model” in “sjPlot” v. 2.8.11 r package (Lüdecke 2024). Dots represent the observed data.

The acoustic analyses confirm clustering of songs based on their population of origin (Fig. 1a). “Dissimilarity to local centroid” values showed little overlap between local and foreign populations: local songs, 0.0087 to 0.0319; Spanish songs, 0.0255 to 0.0639; Dutch songs, 0.0321 to 0.0494 (Fig. 1b). Pied flycatcher males were observed responding to played-back songs in at least one of the paired nest boxes in 54 out of 97 conducted trials (55.7%). Out of these 54 trials, both boxes were visited in 23 trials, whereas only one box was visited in the rest. Only 33.8% of the males that visited a nest box showed aggressive behavior toward the dummy, supporting the independent analyses of the 2 behavioral variables. Unsurprisingly, whether or not an experimenter observed a male around the nest box prior to the start of the playback had a significant effect on the amount of time males were observed during playback (likelihood-ratio test: χ^2^ = 8.97, *P* = 0.0027) (Table 1). After accounting for the effect of male presence, as predicted, the similarity of a given playback to the typical local song had a significant effect on the time a bird spent visiting the nest box (likelihood-ratio test: χ^2^ = 4.46, *P* = 0.034). Thus, males responded significantly longer to playbacks that were more similar to the centroid of the local population, i.e. songs with lower “Dissimilarity to local centroid” scores (Fig. 2). Regarding aggressive responses toward the wooden dummy, these were not affected by whether a male was seen around the nest box prior to start the playback. However, males behaved more aggressive as the played-back songs were more similar to the local centroid (likelihood-ratio test: χ^2^ = 6.23, *P* = 0.0125) (Table 2 and Figure 3).

**Table 1.** Summary results of Generalized Linear Mixed Model showing the effect of “Dissimilarity to the local centroid” and “Male seen” on “Time present” of males during playback experiments.

	Estimate ± SE	Z	*P* value
Intercept	6.133 ± 0.534	11.476	<0.0001
Dissimilarity to local centroid	−28.456 ± 13.51	−2.106	0.035*
Male seen (Yes)	0.785 ± 0.264	2.97	0.003**
Year (2023)	0.111 ± 0.255	0.438	0.661

Asterisks indicate the level of statistical significance: ***, <0.001; **, <0.01; *, ≤0.05

**Table 2.** Summary results of Generalized Linear Mixed Model showing the effect of “Dissimilarity to the local centroid” and “Male seen” on “Aggressiveness” (number of pecks on dummy) of males during playback experiments.

	Estimate ± SE	Z	*P* value
Intercept	4.116 ± 1.059	3.886	0.0001
Dissimilarity to local centroid	−58.467 ± 24.743	−2.363	0.018*
Male seen (Yes)	0.69 ± 0.57	1.21	0.226
Year (2023)	−1.049 ± 0.494	−2.124	0.034*

Asterisks indicate the level of statistical significance: ***, <0.001; **, <0.01; *, ≤0.05

## Discussion

We found that the strength of male pied flycatcher responses to songs depends on their similarity of those typical of their home population. These findings are consistent with the hypothesis that divergence in sexual signals promotes incipient reproductive barriers across populations of oscine birds. Here, we will discuss (1) how song discrimination arises with song complexity, (2) the role of experience in determining adult song responses, and (3) the potential consequences of discrimination for putative interactions between populations.

Previous theoretical work has suggested that song-sharing and the formation of song dialects and, by extension divergence in song discrimination, should be challenged by song complexity (Williams and Slater 1990). Indeed, song discrimination has primarily been demonstrated in species having relatively simple song (Parker et al. 2018). Our results here demonstrate that, at least for pied flycatchers, relatively high complexity, on the order of 50 syllable types per male, is not a hindrance for the emergence of population-level song discrimination. Importantly, we do not argue that the population-level song differences we demonstrate in pied flycatchers represent dialects, which are typically defined as sharp turnover of syllable- and/or song-types over significantly smaller geographic scales (100s of km) than those we explore here in flycatchers (1,000 to 2,500 km). Instead, our measure of song dissimilarity compared playback recordings with the centroid of our focal population using a broad range of spectral features. A previous study using a similar approach found that more than 80% of songs from 6 European populations could be correctly classified to the population level (Wheatcroft et al. 2022). This suggests that a range of spectral features, rather than a steep geographic cline in a particular syllable- or song-type, are likely to be utilized by adult pied flycatcher males to adjust their responses to song playbacks.

Based on typical natal dispersal distances, the pied flycatcher males included in our study are likely to have been born in and, therefore, likely to have been exposed to songs from a 50-km radius around our focal population, Tovetorp (Thomson et al. 2003). Some studies explored the role of social learning during upbringing in pied flycatcher song acquisition. Cross-fostered chicks included heterospecific song types in their songs as adults (Eriksen et al. 2009). A similar effect was observed when translocating pied flycatcher eggs across populations, where their songs sounded more similar to the foster than to the ancestral population (Rajan et al. 2024). Moreover, there is evidence that young flycatchers in our population are exposed to adult songs while in the nestbox (see Supplementary material in Wheatcroft et al. 2022). As a result, discrimination in favor of local-like songs is likely to arise in large part due to experience in combination with any innate predispositions to learn local songs (Wheatcroft et al. 2022; Rajan et al. 2024). Because we lack knowledge of the natal origin of the males included in our study, we cannot rule out the possibility that their experience early in life consisted of non-Tovetorp songs. Assuming that song divergence is at least in part dependent on geographic distance, it is likely that even foreign-born males would have been exposed to songs more similar to those from Tovetorp than those from the very distant, foreign populations used in our study. Moreover, pied flycatcher males have been suggested to learn and produce song syllables throughout their lives (Eriksen et al. 2011), meaning that, even if the adult males in our experiment originated in another location, they would have had ample opportunity to learn about Tovetorp-typical songs. Put together, we suggest that song discrimination demonstrated in our study is likely to arise largely through experience with local songs. Due to the large population turnover in our population, we can exclude the possibility that the local songs played back derived from individuals familiar to the responding birds. Thus, we can conclude that stronger responses to the local playbacks are due to the overall acoustic characteristics of the songs rather than as a byproduct of neighbor–stranger recognition.

Like males, females songbirds acquire their song preferences through song experience early in life (Riebel 2000; Fujii et al. 2021). Pied flycatcher female dispersal is broadly similar to that in males, typically less than 50 km (Chernetsov et al. 2006; Both et al. 2012; Sirkiä et al. 2013) and, assuming moderate song divergence by distance, female preferences for local songs likely primarily arise through a combination of juvenile and post-natal experience during the first year. Stronger responses to local songs in female birds are likely to maintain local song culture (Lachlan et al. 2014) and suggest the potential for incipient reproductive barriers across populations (e.g. Danner et al. 2011). The degree to which song divergence drives reproductive divergence has been under debate for over 40 yr (Baker and Cunningham 1985; Macdougall-Shackleton and Macdougall-Shackleton 2001; Ruegg et al. 2006; Yoktan et al. 2011; Lipshutz et al. 2017). Interestingly, despite variation in population genetic differentiation among the populations included in our study (0.002 Fst, Netherlands to Lund, Sweden; 0.022 to 0.023, Spain to Lund, Sweden; Lehtonen et al. 2009), the songs from the foreign populations are equally dissimilar to those of our focal population at Tovetorp (see Fig. 1b), suggesting a complex relationship between divergence in songs and genomes. This may align with some examples where a larger divergence in song than in genomes had been reported (Soha 2004; Robin et al. 2011; Searfoss et al. 2020).

To conclude, our results demonstrate population-level discrimination in a species with a comparatively high degree of song complexity. This raises the possibility that relevant population variation in songs is more widespread than is currently recognized. This is in line with a recent study arguing that “cryptic” dialects in zebra finches, identified using machine learning, are relevant to mate choice (Wang et al. 2022). Our study demonstrated song discrimination in a single population. Based on previous results in nestlings (Wheatcroft et al. 2022), we argue that other pied flycatcher populations are likely to exhibit a similar degree of population-specific song responses. We suggest that future field playback studies in additional populations as well as over smaller spatial scales (100, 50, or 5 km) would help to further define the role of geographical isolation in song discrimination in species with complex songs.

## Data Availability

Analyses reported in this article can be reproduced using the data provided by Gallego-Abenza et al. (2024).
